# Interlimb strength asymmetry is task-specific: moderate reliability but inconsistent limb dominance in multi-joint dynamometry

**DOI:** 10.3389/fspor.2026.1836014

**Published:** 2026-05-25

**Authors:** Manfred Zöger, Alfred Nimmerichter, Arnold Baca, Klaus Wirth

**Affiliations:** 1 Training and Sports Sciences, University of Applied Sciences Wiener Neustadt, Wiener Neustadt, Austria; 2 Centre for Sport Science and University Sports, University of Vienna, Wien, Austria; 3 Doctoral School of Pharmaceutical, Nutritional and Sport Sciences, University of Vienna, Wien, Austria

**Keywords:** isokinetic leg press, isometric leg press, limb dominance, lower-limb strength asymmetry, multi-Joint dynamometry, test-retest reliability

## Abstract

**Background:**

Interlimb strength asymmetry is widely used in sports medicine and performance diagnostics, yet the reliability of asymmetry measures in multi-joint dynamometry, their velocity specificity, and the consistency of limb dominance remain unclear. This study examined the test-retest reliability of interlimb strength asymmetry derived from unilateral multi-joint isometric and isokinetic dynamometry and investigated whether limb dominance remains consistent across different knee angles and contraction velocities in recreationally active adults.

**Methods:**

Twenty-five recreationally active adults, engaging in 150–300 min of moderate-to-vigorous physical activity per week and full-body resistance training without participation in elite-level sports, performed unilateral leg press assessments at two isometric knee angles (100° and 140°) and two isokinetic velocities (30 and 600 mm·s⁻¹) in two separate sessions. Interlimb strength asymmetry was calculated from peak force. Directional asymmetry was determined by assigning a positive or negative sign to represent right- or left-leg dominance, respectively. Systematic differences between sessions were evaluated using paired-sample t-test. Reliability was assessed using single-measure intraclass correlation coefficients (ICC), standard error of measurement (SEM), minimum detectable change (MDC), kappa coefficients, and Bland–Altman analysis.

**Results:**

No significant systematic differences between sessions were detected (*p* > 0.05), and mean asymmetry values were small (range −3.5% to 1.1%). Relative reliability ranged from moderate to good (ICC = 0.55–0.84), whereas absolute reliability indicated substantial variability (SEM = 2.9%–7.5%; MDC = 8.1–20.8%). Limb dominance showed fair to substantial inter-session agreement (kappa = 0.24–0.65), with 16% of participants demonstrating consistent dominance across all conditions and sessions.

**Conclusions:**

These findings suggest that interlimb strength asymmetry in multi-joint dynamometry is task-specific, exhibiting considerable individual variability and limited stability of dominance. As measurement error frequently approached or exceeded commonly cited clinical thresholds (e.g., 10%–15%), practitioners should interpret asymmetry values cautiously and avoid relying on universal cutoffs for return-to-sport or performance decision-making.

## 1 Introduction

Lower-limb strength imbalances are widely recognized as significant risk factors for both primary and secondary musculoskeletal injuries (1, 2). Specifically, asymmetries are frequently implicated in hamstring strains and knee injuries, most notably anterior cruciate ligament ruptures (1, 3–5). Consequently, the assessment of muscular asymmetry has become a cornerstone of clinical practice, serving as a key metric for return-to-sport clearance and the discharge of patients from rehabilitation (6, 7). Beyond injury prevention, emerging evidence suggests that pronounced strength asymmetries may also impair athletic performance (8–11), further highlighting the need for accurate assessment.

In clinical and performance settings, a deficit of 10%–15% is often cited as the threshold for concern (12–14). However, these benchmarks remain controversial and are largely based on anecdotal evidence or traditional practice rather than robust, evidence-based data (12, 15). As a result, there is significant ambiguity regarding if and at what point an asymmetry becomes clinically or functionally problematic (12, 15). This lack of clarity has led to growing critical discourse in recent literature concerning the validity of universal thresholds (13, 16, 17). To address this, recent research has employed data-driven approaches, such as classification and regression tree analysis, to identify task-specific thresholds, demonstrating that critical values are highly dependent on the specific movement (18).

One primary driver of these discrepancies may be the variation in testing methodologies. Current diagnostics typically utilize dynamometric assessments (predominantly isokinetic) or functional field tests, such as various jump protocols (19, 20). However, the generalizability of these findings is questionable and research suggests that asymmetry is highly task-, metric- and time-specific (21–26). Furthermore, asymmetry appears to be a multidimensional construct. For instance, functional asymmetries have shown no meaningful association with lean mass imbalances, suggesting that morphological and functional metrics capture distinct aspects of asymmetry (27). Notably, lower limb measures have demonstrated greater variability compared to upper limb assessments, further highlighting the region-specific nature of these asymmetries (24, 27). While muscular imbalances are a vital metric, there is currently no consensus on the optimal diagnostic approach or definitive critical thresholds. This is consistent with observations that monitoring strategies often lack standardization across different sporting contexts (28). Recent literature underscores that reporting reliability is a fundamental requirement in asymmetry research (19). However, substantial challenges remain regarding the stability of these measures. While functional tests often show acceptable relative reliability, their absolute reliability and the consistency of limb dominance frequently exhibit poor stability across sessions (15, 19). This inconsistency complicates the interpretation of how asymmetries relate to performance, as highlighted in recent systematic reviews (11). While Bishop et al. (29) focused on functional protocols and others, such as Impellizzeri et al. (30) have examined isokinetic single-joint leg extensions, data regarding multi-joint dynamometry assessments remains scarce.

To date, to the best of the authors’ knowledge, no study has specifically investigated the reliability and consistency of strength asymmetries derived from isokinetic and isometric multi-joint dynamometry testing. This represents a significant gap in the literature, as multi-joint movements may more closely reflect the demands of functional tasks (31) while maintaining the precision of laboratory-based testing. This study aims to address this gap by investigating the following research questions: (1) What is the test-retest reliability of interlimb strength asymmetry during multi-joint isometric and isokinetic dynamometry? and (2) How consistent is limb dominance across different knee angles, angular velocities, and testing sessions in healthy, physically active adults? Given the paucity of research on multi-joint dynamometry in this context, the study followed an explorative approach regarding the consistency of limb dominance. However, based on existing findings from functional tests (29), we hypothesized that while the magnitude of asymmetry might show moderate reliability, the consistency of limb dominance would show significant variability across sessions.

## 2 Materials and methods

### 2.1 Subjects

A total of 27 participants were initially recruited for the study, using a convenience sampling approach via institutional email lists and internal communication boards at the University of Applied Sciences Wiener Neustadt. Of these, 25 participants completed the full experimental protocol. Two individuals withdrew during the course of the investigation due to scheduling conflicts and time-related constraints. Consequently, the final sample consisted of 25 subjects (mean ± SD): stature 178.4 ± 8.5 cm; body mass 75.8 ± 9.8 kg; age 26.1 ± 6.4 years. To be eligible for participation, indiviuals were required to engage in 150–300 min of moderate-to-vigorous physical activity per week, consistent with current physical activity recommendations (32), and to participate regularly in recreational sports activities and full-body resistance training without involvement in professional or elite-level athletic training. Exclusion criteria included any self-reported musculoskeletal injury to the lower extremities that would have needed clinical treatment or could impair functional performance testing. Regarding lateral preference, 23 participants were right-leg dominant, defined as the preferred limb for kicking a ball (33). While it is acknowledged that limb preference for kicking is task-specific and may not necessarily align with strength-based dominance (19, 34), this metric was collected solely to provide a descriptive profile of the sample’s lateral preference prior to functional testing. To ensure standardized testing conditions, participants were strictly instructed to adhere to the following pre-trial requirements: abstain from strenuous physical activity for 48 h prior to each session; refrain from caffeine consumption for at least 12 h before testing; abstain from food for 3 h immediately preceding each trial. The study was conducted in accordance with the Declaration of Helsinki (35) and was approved by the local research ethics board at the University of Applied Sciences Wiener Neustadt (approval no. RB20210405013). Prior to commencement, all participants were fully briefed on the potential risks and benefits associated with the study. Each participant provided written informed consent and was explicitly notified of their right to withdraw from the investigation at any time without providing a reason.

### 2.2 Instruments

**Figure 1:**
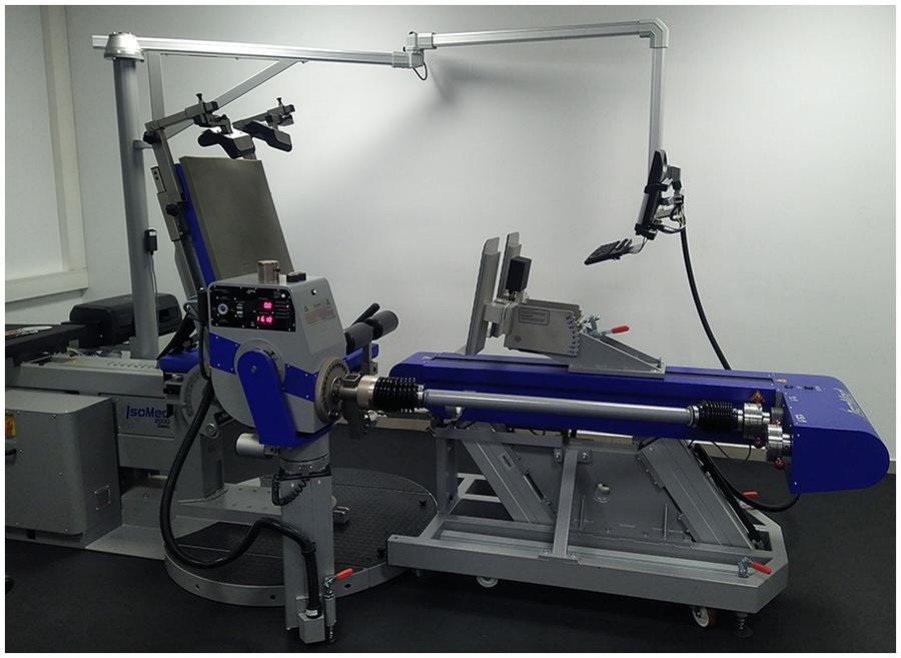
The Iso Med 2000 device converted into a motor-driven leg press using the manufacturer’s athletic linear module.

All tests were conducted using an IsoMed 2000 dynamometer (D. & R. Ferstl GmbH, Hemau, Germany) in combination with the manufacturer’s athletic linear module, which converts the dynamometer into a motor-driven leg press. Strain gauge force sensors integrated into the footrest permit independent measurement of the applied force for the left and right limbs. For the purposes of this study, gear I of the drive shaft was utilized (ratio 1:1; maximum linear velocity of 800 mm·s^−1^, and a maximum force of 8,850 N) (36). The setup is illustrated in Figure 1. Prior to each testing session, the device was calibrated in accordance with the manufacturer’s instructions. Data were recorded at a sampling rate of 200 Hz using the integrated computer software IsoMed analyze (version SP3-i51).

### 2.3 Procedures

In accordance with established recommendations (36–39), all subjects underwent a familiarization session prior to the experimental tasks to become acquainted with the equipment, the isokinetic exercise modality, and the testing protocol. This session was identical to the subsequent experimental sessions, ensuring that participants were fully accustomed to the characteristics of isokinetic exercise before the main data collection. Sessions were separated by 72–96 h to ensure sufficient recovery. To minimize the influence of diurnal variations, sessions for each subject were conducted at the same time of day (± 1.5 h). All sessions were supervised by the same researcher to eliminate inter-tester variability. Participants were instructed to wear shoes with a hard rubber outsole and no additional cushioning, maintaining the same footwear for all sessions.

Each session commenced with a 10 min standardized general warm-up, consisting of 10 min cycling on a stationary ergometer at a submaximal intensity of 1.5 W per kg bodyweight and a cadence of around 70 rpm. Following this general warm-up, participants were seated in the adjustable dynamometer chair with the backrest positioned at 75°. The footrest was set at an inclination of 10° towards plantar flexion of the ankle. To ensure stability and minimize errant body movements, adjustable seat belts and pads were firmly secured across shoulders, thorax, and hips. In addition, the subjects were instructed to grasp the side handles during testing.

Range of motion and velocity served as the primary input parameters (40). A mechanical handheld goniometer was utilized to set the range of motion to 90–170° (180° representing full extension). Reference points for goniometry included the trochanter major, lateral femoral epicondyle, and lateral malleolus, all identified via palpation. The start and end positions were verified under muscular tension. For safety and to prevent knee hyperextension, the device's popliteal pad was adjusted to preclude full joint extension.

Subjects were positioned with the heel on the designated support area of the footrest. For isometric measurements, the footrest was fixed to achieve knee joint angles of 100° (Iso100) and 140° (Iso140). The positions were individually adjusted using the aforementioned goniometric methods. Integrated software was used to record these settings to guarantee identical positioning across all sessions. During each session, subjects performed unilateral isometric and isokinetic leg extension under four conditions: Iso100, Iso140, and isokinetic movements at translational velocities of 30 mm·s^−1^ and 600 mm·s^−1^. In line with previous research (41), the slower velocity always preceded the faster one. All isokinetic measurements were executed as single-direction, discrete movements (42), with the starting position reached passively. The testing order remained constant: Iso100, Iso140, 30 mm·s^−1^ and 600 mm·s^−1^ (41, 43). Starting leg (left or right) was randomized and counterbalanced across the group, though each subject always started with the same leg in subsequent sessions.

Prior to each condition, participants performed a specific submaximal warm-up consisting of 10 repetitions at approximately 50% of maximum voluntary contraction, followed by 3 repetitions at 80% of maximum voluntary contraction. This was followed by a 3 min recovery period, during which the examiner provided standardized instructions for the upcoming task.

A minimum of three repetitions were completed for each condition. Additional repetitions were performed if the peak force continued to increase; all subjects reached peak force within a maximum of five repetitions. Each repetition was followed by 3 min of passive rest. To ensure maximal effort, subjects received real-time visual feedback via a monitor and consistent, standardized strong verbal encouragement from the examiner.

### 2.4 Interlimb asymmetry calculation

Relative lower limb strength asymmetry has been calculated using various methods (12, 44). In this study, bilateral strength asymmetry (BSA) was calculated according to the formula established by Bishop and colleagues (45):

$$BSA\;(\text{\%})=\frac{\left(stronger\;limb-weaker\;limb\right)}{stronger\;limb}*100$$

A limitation of this formula is that it yields only positive values. When comparing asymmetries across different test modalities for the same subject, the dominant limb may switch. Relying solely on absolute BSA values obscures the direction of dominance, potentially leading to the erroneous conclusion that two modalities yield identical results when they actually indicate opposite dominance of the same magnitude. To address this issue, we followed the approach of Impellizzeri et al. (46) by assigning a directional prefix to the BSA value: positive values indicate a stronger right leg, while negative values indicate a stronger left leg. In the event of a 0% asymmetry, the case would have been excluded from the directional consistency analysis (kappa). However, no cases of exactly 0% asymmetry were observed in the present dataset.

### 2.5 Statistical analysis

Data are expressed as mean ± SD. For each experimental condition, the repetition yielding the highest peak force during leg extension was selected for subsequent analysis, following established protocols for maximal strength assessment (40, 47, 48). These peak force values served as the basis for calculating the BSA (%) using the previously defined formula. The normality of data distribution was assessed via the Shapiro–Wilk test. To account for the directional nature of asymmetries (favoring either the right or left limb), a Kappa coefficient was calculated to evaluate the consistency of limb dominance across test conditions and sessions (49). Kappa values were interpreted according to criteria proposed by Viera and Garrett (50): < 0 = less than chance agreement, 0.01–0.20 = slight agreement, 0.21–0.40 = fair agreement, 0.41–0.60 = moderate agreement, 0.61–0.80 = substantial agreement and 0.81–0.99 = almost perfect agreement. For visualization purposes, representative Sankey diagrams were generated to illustrate changes in limb dominance between sessions within one condition and changes in dominance across conditions within a single-session. Paired-sample t-tests were employed to identify potential systematic bias and determine if asymmetry scores differed significantly between sessions. The magnitude of these differences was quantified using Cohen’s *d* effect sizes, calculated as:

$$d=\frac{M_{diff}}{S_{\;pooled}}$$

where M_diff_ represents the mean difference between repeated measures and S_pooled_ is the pooled SD of the measurements at each time point. Effect sizes were categorized as small (|*d*| ≥ 0.20), medium (|*d*| ≥ 0.50), or large (|*d*| ≥ 0.80) (51–53).

Relative reliability was assessed using a single-measure two-way random-effects intraclass correlation coefficient (ICC), with interpretations based on Koo and Li: values > 0.9 (excellent), 0.75–0.9 (good), 0.5–0.75 (moderate), and < 0.5 (poor). Absolute reliability was also determined by the standard error of measurement (SEM) (54, 55):

$$SEM=SD\;*\;\sqrt{\;1-ICC}$$

The minimum detectable change (MDC) was calculated to establish the threshold for a “real” change beyond measurement error, based on a 95% confidence interval (CI) as described by Weir (55):

$$MDC=SEM\;*\;1.96\;*\;\sqrt{\;2}$$

To assess the level of agreement between sessions, Bland–Altman statistics with ± 95% limits of agreement (LoA) were computed. Corresponding plots were generated to provide a visual representation of individual differences and potential heteroscedasticity.

All statistical procedures were performed using IBM SPSS Statistics, version 31.0.0 (IBM Corp., Armonk, NY, USA). Graphical illustrations were authored in GraphPad Prism, version 11.0.0.84 for Windows (GraphPad Software, San Diego, CA, USA) and Sankey diagrams were generated using RAWGraphs open-source web-based data visualization framework. For all analyses, the alpha level was set at *p* < 0.05.

## 3 Results

Descriptive statistics (mean values ± SD) for peak force and BSA for both sessions, including comparative statistics, are summarized in Table 1. No significant differences were detected between sessions for either peak force or BSA (*p* > 0.05). Mean BSA values ranged from −3.5% to −0.4% in session 1 and from −1.6% to 1.1% in session 2. Cohen’s *d* effect sizes for inter-session BSA variations ranged from 0.03 to 0.24, representing negligible to small effects.

**Table 1:** Mean ± SD for peak force and bilateral strength asymmetry in session 1 and session 2 as well as *p*-values and Cohen’s *d* effect size for comparison of sessions.

Condition	Session 1			Session 2			PF main effect	BSA main effect	BSA
	PF (*N*)	PF_rel_ (*N*·kg^−1^)	BSA (%)	PF (*N*)	PF_rel_ (*N*·kg^−1^)	BSA (%)	*p*-value	*p*-value	ES
Iso100 R	1695.9 ± 518.6	22.5 ± 7.6	−0,4 ± 8.2	1723.6 ± 553.3	22.9 ± 8.0	−0.1 ± 10.1	0.27	0.82	0.03
Iso100 L	1710.8 ± 530.2	22.6 ± 6.9		1721.5 ± 507.0	22.7 ± 6.3		0.67		
Iso140 R	2871.1 ± 796.0	37.7 ± 8.0	−3.5 ± 11.1	2915.7 ± 737.9	38.4 ± 8.0	−1.6 ± 12.1	0.48	0.38	0.16
Iso140 L	2986.1 ± 838.5	39.2 ± 8.9		2996.1 ± 899.4	39.3 ± 9.5		0.91		
30 mm·s^−1^ R	2559.2 ± 582.2	33.7 ± 5.8	−2.3 ± 11.7	2593.9 ± 600.8	34.1 ± 5.7	−0.3 ± 10.7	0.12	0.43	0.18
30 mm·s^−1^ L	2626.8 ± 638.4	34.6 ± 6.3		2602.8 ± 635.8	34.1 ± 6.0		0.74		
600 mm·s^−1^ R	1514.1 ± 339.1	19.9 ± 2.8	−0.6 ± 7.9	1536.4 ± 355.1	20.1 ± 3.0	1.1 ± 6.9	0.22	0.11	0.24
600 mm·s^−1^ L	1524.1 ± 342.6	20.0 ± 2.5		1512.5 ± 325,4	19.8 ± 2.5		0.45		

BSA, bilateral strength asymmetry; PF, peak force; N, newton; PF_rel_, peak force normalized per kg body mass; ES, effect size; Iso100, isometric 100° knee angle; Iso140, isometric 140° knee angle; R, right leg; L, left leg.

**Figure 2:**
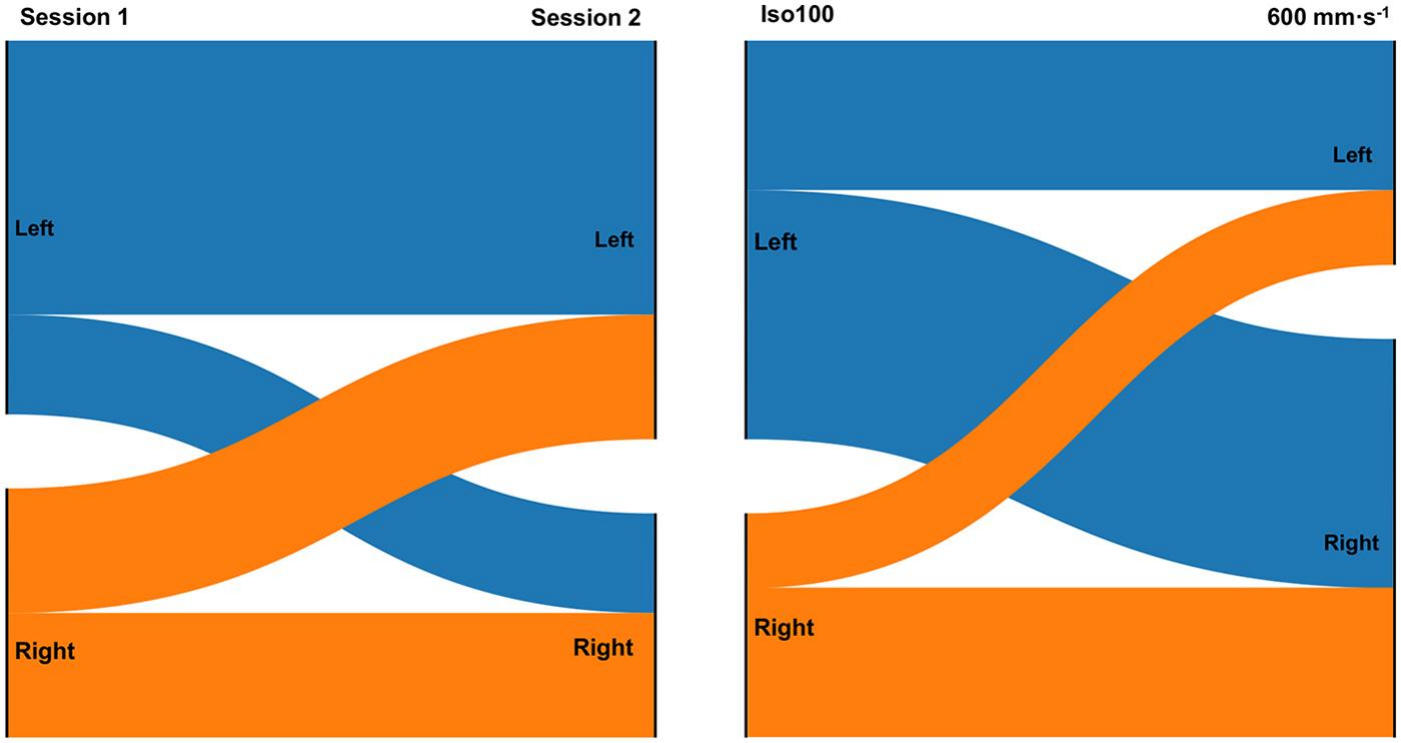
Sankey diagrams illustrating changes in limb dominance; (left panel) Dominance transitions between Session 1 and Session 2 for the Iso140 condition. (right panel) Dominance transitions between the Iso100 and isokinetic 600 mm·s^−1^ conditions within Session 2.

Table 2 summarizes consistency in limb dominance across sessions. Seven participants (28%) exhibited stable limb dominance across both sessions within the same condition; of these, three (12%) showed a reversal of limb dominance in at least one other condition. Consequently, only four subjects (16%) demonstrated consistent limb dominance across all four conditions and in both testing sessions. To illustrate changes in limb dominance, representative Sankey diagrams were created (Figure 2). The Iso140 condition was selected to visualize dominance changes between sessions because it showed the largest number of directional shifts. Additionally, a Sankey diagram comparing the Iso100 and isokinetic 600 mm·s^−1^ conditions within Session 2 was generated to illustrate task-related differences in dominance.

**Table 2:** Inter-session consistency of bilateral strength asymmetry and kappa coefficients between session 1 and session 2. Descriptive levels of agreement indicate the stability of limb dominance across separate testing days.

Condition	Subjects (n, %) with a BSA change > 10% between S1-S2	Subjects (n, %) with a reversal of limb dominance between S1-S2	Subjects (n, %) with a reversal of limb dominance between S1-S2 including a BSA change > 10%	Kappa coefficient	Descriptor
Iso100	4 (16%)	4 (16%)	2 (8%)	0.65	Substantial
Iso140	10 (40%)	9 (36%)	5 (20%)	0.24	Fair
30 mm·s^−1^	9 (36%)	5 (20%)	2 (8%)	0.60	Moderate
600 mm·s^−1^	2 (8%)	7 (28%)	1 (4%)	0.43	Moderate

BSA – bilateral strength asymmetry, S – session, Iso100 – isometric 100° knee angle, Iso140 – isometric 140° knee angle.

**Figure 3:**
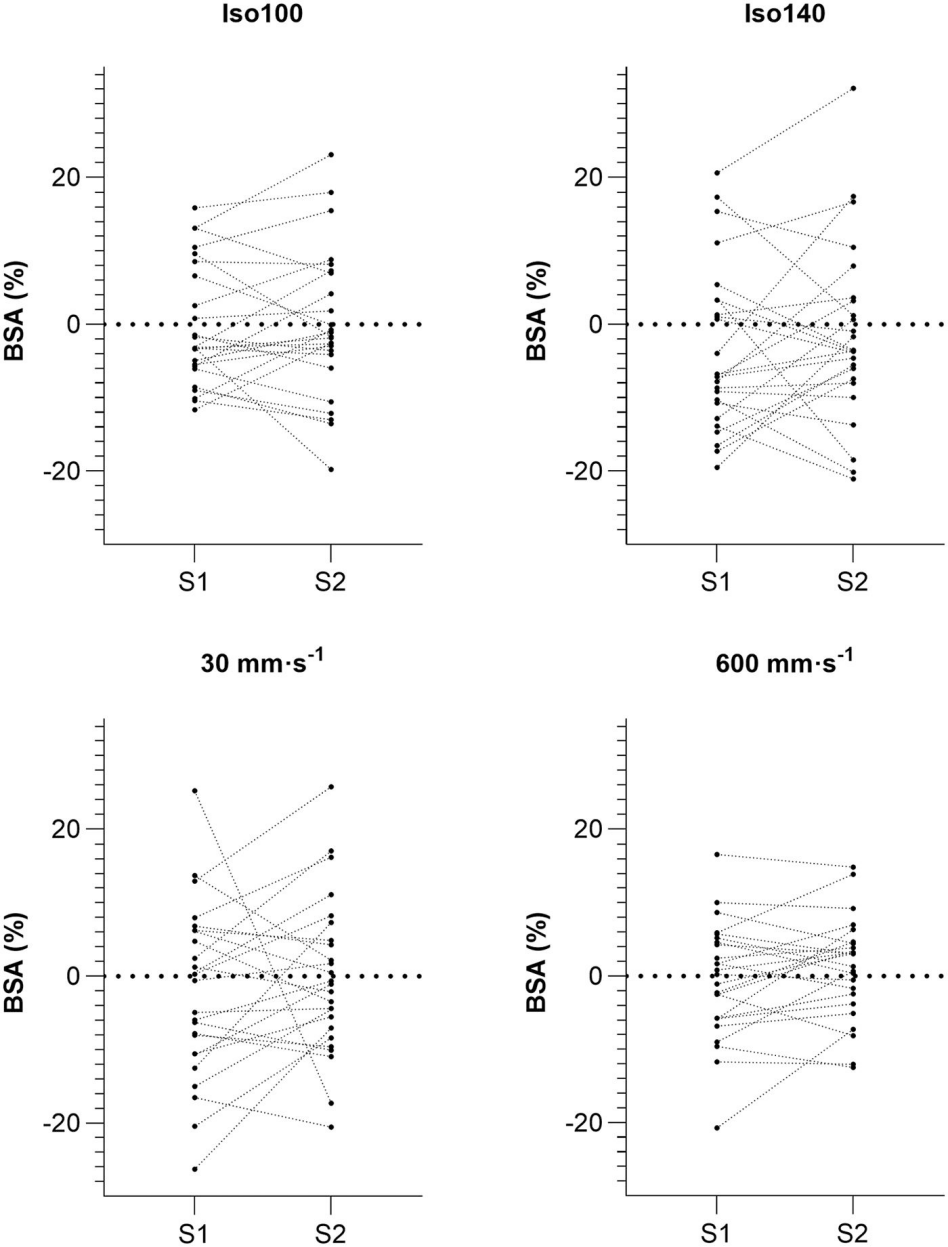
Individual alterations in BSA between S1 and S2 for Iso100, Iso140, 30 mm·s^−1^ and 600 mm·s^−1^; BSA, bilateral strength asymmetry; S, session; Iso100, isometric extension 100° knee angle; Iso140, isometric extension 140° knee angle.

**Figure 4:**
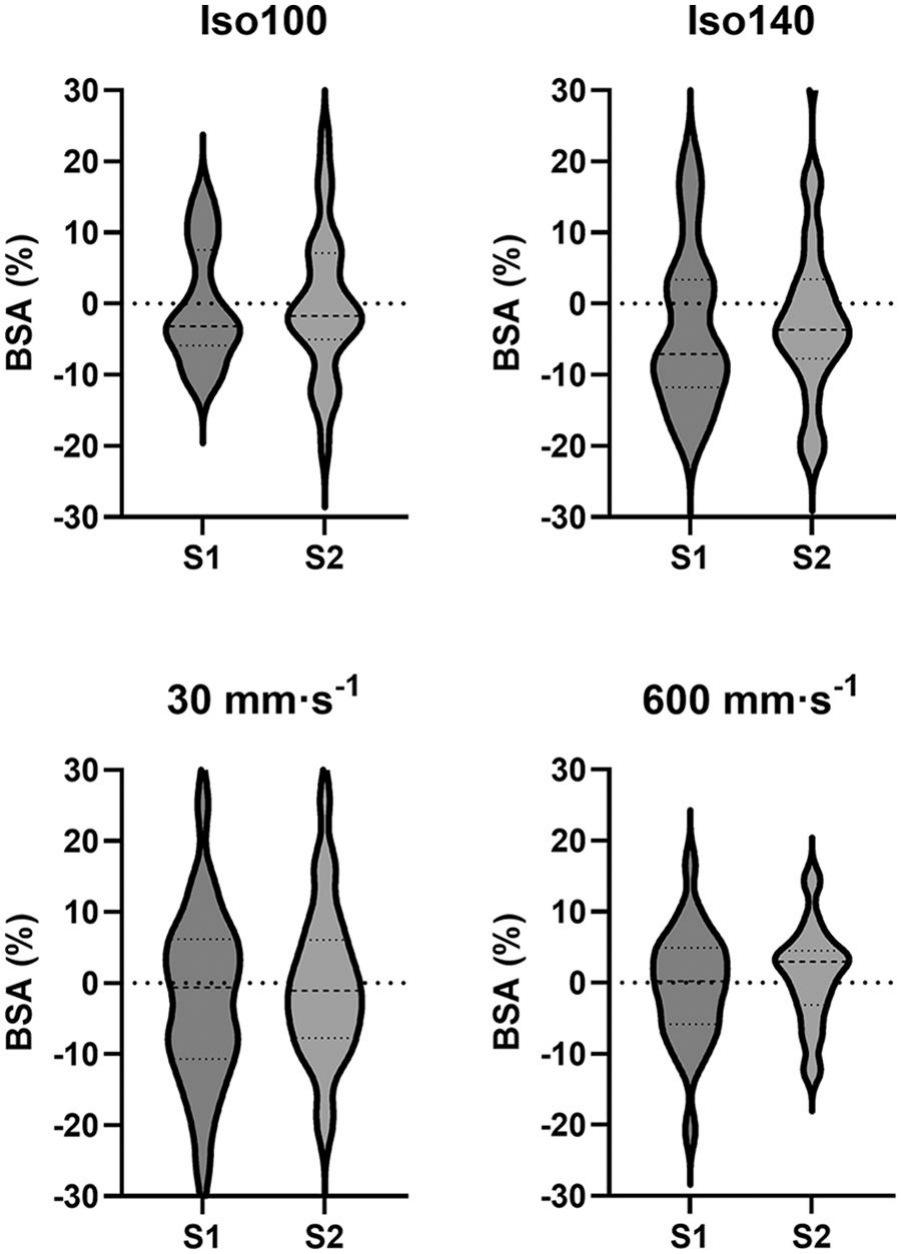
Violin-plots – distribution of BSA for S1 and S2 for Iso100, Iso140, 30 mm·s^−1^ and 600 mm·s^−1^; BSA, bilateral strength asymmetry; S, session; Iso100, isometric extension 100° knee angle; Iso140, isometric extension 140° knee angle.

Kappa coefficients for inter-session comparison indicated fair to substantial agreement (range = 0.24–0.65). Intra-session agreement (Table 3) was lower, with session 1 yielding slight to moderate agreement (0.11–0.44) and session 2 remaining relatively stable with slight to moderate agreement (0.04–0.46). Individual shifts in BSA between sessions are visualized in Figure 3, while the distribution of BSA across conditions and sessions is illustrated via violin plots in Figure 4.

**Table 3:** Kappa coefficient including descriptive level of agreement showing how consistently asymmetry favors the same leg within sessions 1 and 2, respectively.

Session 1		Kappa coefficient	Descriptor
Iso100	Iso140	0.24	Fair
Iso100	30 mm·s^−1^	0.11	Slight
Iso100	600 mm·s^−1^	0.37	Fair
Iso140	30 mm·s^−1^	0.36	Fair
Iso140	600 mm·s^−1^	0.29	Fair
30 mm·s^−1^	600 mm·s^−1^	0.44	Moderate

Session 2		Kappa coefficient	Descriptor
Iso100	Iso140	0.31	Fair
Iso100	30 mm·s^−1^	0.34	Fair
Iso100	600 mm·s^−1^	0.04	Slight
Iso140	30 mm·s^−1^	0.34	Fair
Iso140	600 mm·s^−1^	0.04	Slight
30 mm·s^−1^	600 mm·s^−1^	0.46	Moderate

Iso100, isometric 100° knee angle; Iso140, isometric 140° knee angle.

Reliability metrics are presented in Table 4. Relative reliability, as assessed by ICC, ranged from 0.55 to 0.84, indicating moderate to good reliability (95% CI −0.03–0.93). Regarding absolute reliability, SEM values ranged from 2.9% to 7.5%. The MDC, established to identify the threshold for true change beyond measurement error, was calculated to be between 8.1% and 20.8%.

**Table 4:** Relative and absolute reliability statistics for bilateral strength asymmetry including intraclass correlation coefficient and 95% confidence interval, standard error of measurement, minimum detectable change and bland–altman statistics including bias (±SD) and 95% limits of agreement for comparison of session 1 and session 2.

Condition	ICC	ICC 95% CI	SEM	MDC	Bias (± SD)	95% LoA
Iso100	0.84	0.64–0.93	3.6	10.0	−0.3 (± 6.9)	−13.8–13.2
Iso140	0.73	0.39–0.88	6.0	16.6	−1.9 (± 10.8)	−23.0–19.2
30 mm·s^−1^	0.55	−0.03–0.80	7.5	20.8	−2.0 (± 12.6)	−26.7–22.6
600 mm·s^−1^	0.84	0.65–0.93	2.9	8.1	−1.8 (± 5.3)	−12.2–8.6

ICC, intraclass correlation coefficient; CI, confidence interval; SEM, standard error of measurement; MDC, minimum detectable change; SD, standard deviation; LoA, limits of agreement; Iso100, isometric 100° knee angle; Iso140, isometric 140° knee angle.

**Figure 5:**
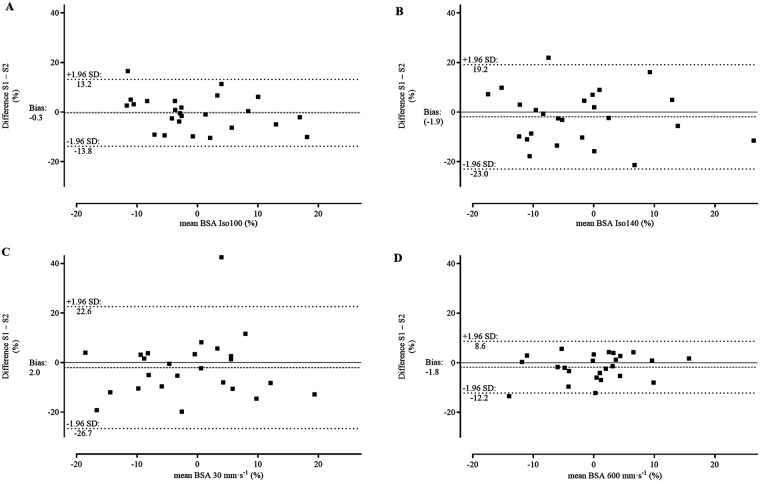
Bland–altman plots – differences between BSA in S1 and S2 plotted against the means of S1 and S2. Plots for: **(A)** Isometric extension at 100° knee angle; **(B)** Isometric extension at 140° knee angle; **(C)** Isokinetic extension at 30 mm·s^−1^ and **(D)** Isometric extension at 600 mm·s^−1^; BSA, bilateral strength asymmetry; S, session; Iso100, isometric extension 100° knee angle; Iso140, isometric extension 140° knee angle.

Inter-session agreement was further evaluated using Bland–Altman plots (Figure 5), which depict subject-specific differences relative to session means. The systematic bias between sessions was low, ranging from −2.0% to −0.3%, though the 95% LoA demonstrated a broader spread from −26.7% to 22.6%.

## 4 Discussion

The primary objective of this study was to evaluate the reliability and consistency of lower-limb strength asymmetries using multi-joint isokinetic and isometric dynamometry across various isometric angles and isokinetic velocities. While traditional clinical practice frequently utilizes fixed thresholds for identifying problematic imbalances (12, 22, 56), the findings of this study suggest that interlimb asymmetry is a highly volatile metric at the individual level, despite appearing stable in group-level mean data. The results indicate that there were no significant systematic differences in BSA between testing sessions (*p* > 0.05). However, the individual consistency of limb dominance was found to be remarkably low. Only 16% of participants demonstrated a consistent dominant limb across all test conditions and sessions. This indicates that the direction of asymmetry is neither stable nor generalizable across different testing modalities, further supporting the notion that asymmetry is highly task-specific (21–23). The observed ICC values suggest moderate to good relative reliability for asymmetry scores. However, the associated 95% CI were relatively wide, with some lower bounds approaching or even crossing zero, indicating substantial uncertainty in the stability of these estimates. This could be influenced by the relatively small sample size and suggests that the true reliability may be lower than the point estimates imply. Moreover, relative reliability measures like the ICC do not account for the absolute measurement error necessary to guide clinical decision-making. Therefore, the interpretation of interlimb asymmetry should be anchored to the MDC rather than arbitrary fixed cut-offs. This distinction becomes evident when considering our MDC values (ranging up to 20.8%), which frequently exceeded the commonly cited 10%–15% thresholds. This suggests that in many testing conditions, what is often labeled as a clinically relevant asymmetry may actually fall within the range of inherent measurement noise. However, a more detailed analysis reveals that this measurement error was not uniform across all testing modalities. While the slow isokinetic condition exhibited the highest MDC (20.8%), which exceeds common clinical requirements, other conditions demonstrated considerably greater stability. Specifically, the fast isokinetic condition and Iso100 yielded MDC values of 8.1% and 10.0%, respectively. These values fall within or near the 10%–15% threshold for clinical adequacy, suggesting that the reliability of multi-joint dynamometry is highly task-specific. Faster or isometric contraction modes may therefore provide more stable metrics for individual monitoring than slow-velocity protocols, which seem to be more susceptible to neuromuscular ‘noise’.

A notable observation of this study is the frequent occurrence of limb dominance reversal. The kappa coefficients for inter-session agreement (0.24–0.65) and intra-session agreement (0.04–0.46) suggest that the limb identified as stronger in one session or at one specific velocity is frequently the weaker limb in another. The Bland–Altman analyses provide further support for this interpretation. While the presence of systematic bias between sessions was found to be negligible, with bias ranging from −2.0% to −0.3%, the relatively wide LoA from −26.7% to 22.6%, which suggests considerable inter-individual dispersion. Consequently, even in the absence of systematic bias, the individual-level agreement was found to be limited. These results align with previous research suggesting that asymmetry is not only task-specific but also highly sensitive to the mechanical constraints of the test, such as velocity and joint angle (21–23). From a practical standpoint, this finding suggests that single asymmetry measurements should be interpreted with caution when guiding high-stakes decisions, such as return-to-sport clearance. This is particularly relevant as our results demonstrate that the measurement error and the inconsistency of limb dominance may exceed the magnitude of the asymmetries being monitored. Therefore, unless a testing protocol demonstrates high reliability and stability in the direction of asymmetry, its utility in clinical decision-making should be considered with caution.

The present findings align with previous literature emphasizing the context-specific nature of asymmetry. For example, Bishop et al. (21) highlighted the complexity of asymmetry and noted that functional assessments frequently exhibited fluctuating dominance. The authors of the study computed Within-Session kappa coefficients for the purpose of comparison of unilateral isometric squats with single-leg countermovement jumps and single-leg broad jumps, finding values as low as −0.34–0.05. The intra-session kappa values in our study (0.04–0.46) indicate that, even within the same testing modality (that is multi-joint dynamometry), alterations in velocity or angles sufficiently modify recruitment patterns to effect a shift in the asymmetry profile. It is noteworthy that the between-session Kappa value of up to 0.65 exhibited in our study was comparable to the 0.64 reported by Bishop et al. (29) for Isometric Squat peak force. This finding suggests that, while dominance may exhibit significant variability across different tasks, there is a tendency towards slightly higher and fair to substantial agreement when the exact same task is repeated. However, this stability appears to be highly time-dependent. Longitudinal evidence in youth athletes has shown that both the magnitude and direction of asymmetry in the lower extremity are remarkably unstable over several years (24). Furthermore, even within shorter timeframes, acute factors such as muscle fatigue can significantly alter asymmetry profiles, as demonstrated by fluctuating jump asymmetries in the 72 h following a fatiguing protocol (25). Taken together with our findings, this suggests that asymmetry is not a fixed trait but a dynamic state that is sensitive to both mechanical constraints and temporal factors.

Bailey et al. (57) observed that weaker subjects tend to exhibit greater asymmetries than stronger ones, concluding that absolute strength levels could play a significant role in imbalance. In our cohort of recreationally active individuals, the mean BSA values were found to be comparatively low (near 0%), while the individual variance was found to be high. Applying Bailey’s reasoning, the elevated MDC values (up to 20.8%) in our study may be partially explained by the recreational status of the participants. It is possible that highly trained athletes with greater absolute strength would demonstrate more stable asymmetry profiles and lower MDC values, as their motor patterns are more deeply ingrained.

As documented by Impellizzeri et al. (46), a significant correlation (r = 0.83) was previously established between BSA derived from a vertical jump force test and isometric leg press peak force. While this suggests some cross-test association, correlation does not imply agreement in magnitude at the individual level. The present results demonstrate that even within the same dynamometer and movement pattern, limb dominance frequently shifted depending on angle or velocity. Consequently, extrapolating asymmetry values across different modalities may be problematic.

A critical observation derived from the analysis of our data is the moderate reliability of BSA, accompanied by substantial LoA and MDC. The Bland–Altman plots (Figure 4) revealed LoA ranging from −26.7% to 22.6%. Consequently, for a single individual, an asymmetry score could fluctuate by over 20% between sessions purely due to measurement error or biological variability. In clinical decision-making contexts, when the MDC is set at 20% and a 15% threshold is utilized to ascertain return-to-sport readiness, there is a high risk of misinterpreting random variation as a substantial clinical deficit.

Collectively, the present findings contribute to the growing body of literature questioning universal asymmetry thresholds (13, 16, 17). If asymmetry is indeed task- and metric-specific, and if measurement error can approach or exceed commonly cited clinical cut-offs, then rigid benchmarks (e.g., 10%–15%) may lack sufficient empirical justification. Crucially, any observed asymmetry that falls within the range of the MDC must be considered measurement noise and is therefore not clinically meaningful. Failing to account for this error risk leads to the misinterpretation of natural variability as a functional deficit, potentially resulting in inappropriate clinical interventions.

While the present study provides novel data on BSA in multi-joint dynamometry, it is important to acknowledge several limitations. First, the sample consisted of recreationally active individuals without injury history. Consequently, the findings may not be applicable to elite athletes or clinical populations, where asymmetries may potentially be more pronounced and persistent. While clinical decisions regarding return-to-sport typically involve injured populations, establishing the inherent variability of asymmetry in a healthy cohort is a fundamental prerequisite. Additionally, while an *a priori* power analysis was not performed due to the lack of comparable pilot data for this specific multi-joint protocol, the sample size of *n* = 25 aligns with established reliability studies in the field (29, 30) and was sufficient to provide 95% CI for reliability metrics. Secondly, although a familiarization session was provided, the complexity of multi-joint isokinetic movements may require more extensive practice and higher consistency would be achieved after a longer period of familiarization.

Future research should prioritize the development and validation of more robust, task-specific asymmetry assessment methods to enhance measurement reliability. Specifically, investigating the longitudinal stability of these metrics is essential to distinguish between inherent biological variability and measurement noise. Establishing procedures with lower MDC values and higher dominance consistency is a necessary prerequisite before these metrics can be effectively implemented in clinical screening or long-term athletic monitoring. This is particularly relevant for future studies in injured populations, which could determine whether the variability observed here reflects a functional characteristic of healthy motor control–where the nervous system employs diverse strategies to accomplish multi-joint tasks or whether it primarily reflects limitations of the testing modality. Studying injured populations, who typically exhibit more constrained or stereotypical movement patterns due to functional deficits, would provide a crucial comparison. If the direction of asymmetry remains unstable even when a clear clinical pathology is present, this would identify the multi-joint dynamometric protocol as the primary source of noise. Conversely, more stable profiles in injured patients would suggest that the variability observed here is indeed a hallmark of non-pathological movement.

### 4.1 Implications

In conclusion, the present study demonstrates that interlimb asymmetry scores derived from multi-joint dynamometry exhibit moderate to good relative reliability. However, this is offset by high absolute measurement error, as evidenced by the large MDC values. Consequently, individual asymmetry is highly volatile, and the observed ‘noise’ often exceeds the magnitude of the asymmetries themselves. This limits the practical utility of single-session multi-joint testing for identifying true physiological asymmetries in individual athletes. The frequent reversal of limb dominance across different velocities and sessions suggests that dominance is a fluid rather than a fixed characteristic in recreational athletes. Furthermore, the elevated MDC values identified (8.1–20.8%) suggest that standard clinical thresholds of 10%–15% should be applied with extreme caution, as they may often fall within the range of measurement error. It is recommended that practitioners prioritize individualized longitudinal tracking over one-time recording assessments. In addition to this, consideration should be given to the specific velocity and joint angle at which the asymmetry is measured, as these factors have been demonstrated to have a fundamental impact on the results.

## Data Availability

The raw data supporting the conclusions of this article will be made available by the authors, without undue reservation.
